# Optimizing diceCT protocols for post-mortem cetacean imaging: diffusible iodine enhancement of a rare narrow-ridged finless porpoise (*Neophocaena asiaeorientalis*) fetal specimen using clinical-grade computed tomography

**DOI:** 10.1186/s12917-025-05191-z

**Published:** 2025-12-13

**Authors:** Adams Hei Long Yuen, Ji-Hyung Park, Kelly Cheuk Wai Chan, Yvonne Miu Ching Chan, Cherry Tsz Ching Poon, Daji Noh, Sooyoung Choi, Byung Yeop Kim, Sang Wha Kim

**Affiliations:** 1 School of Medical and Health Sciences, Tung Wah College, Homantin, Kowloon, Hong Kong Special Administrative Region China; 2 College of Veterinary Medicine and Institute of Veterinary Science, Kangwon National University, Chuncheon, Gangwon Republic of Korea; 3 Department of Surgery, Queen Mary Hospital, Pokfulam, Hong Kong Special Administrative Region China; 4 Department of Marine Industrial and Maritime Police, College of Ocean Science, Jeju National University, Jeju, Republic of Korea

**Keywords:** Post-mortem computed tomography, Diffusible iodine-based contrast enhancement, Contrast-enhanced CT, Narrow-ridged finless porpoise, Neophocaena asiaeorientalis, Cetaceans

## Abstract

**Background:**

Post-mortem imaging has become indispensable in marine mammal research, offering non-destructive alternatives to conventional necropsy. While micro-computed tomography (microCT) provides high resolution for small specimens, its utility in cetacean fetal studies is limited by long scanning time, accessibility, and high cost. Diffusible iodine-based contrast-enhanced CT (diceCT) using clinical-grade scanners presents a potential solution, particularly for endangered species like the narrow-ridged finless porpoise (*Neophocaena asiaeorientalis*) (NRFP), where fetal specimens are exceptionally rare. This study pioneers the application of clinical CT-based diceCT for NRFP fetal imaging, addressing the gap in developmental morphology research.

**Results:**

A 41 cm NRFP fetus was recovered from a stranded pregnant female and preserved for iodine-enhanced CT imaging. The specimen underwent 22 weeks of staining in 1% iodine-ethanol solution, with weekly solution replacement and biweekly clinical CT scans (100 kVp, 300 mAs, 0.3 mm slice thickness) to monitor progression. Complete staining was confirmed by clear radiographic differentiation between adjacent tissue, variance of Hounsfield unit (HU) values of the specimen, and a plateau in the signal-to-noise ratio (SNR) among all scans within the liver. High-resolution 3D reconstructions successfully visualized the gastrointestinal tract, urogenital structures, and cardiovascular network, with preserved spatial relationships.

**Conclusions:**

Clinical-grade diceCT provides a practical alternative to microCT for cetacean fetal imaging by achieving organ-scale resolution. The protocol enabled comprehensive 3D morphological analysis without tissue destruction. The archived dataset and destained specimen offer enduring value for conservation research, education, and method development.

## Background

Post-mortem imaging has become an indispensable tool in marine mammal research, offering non-destructive alternatives to conventional necropsy [1–5]. While computed tomography (CT) has revolutionized specimen analysis, imaging of small specimens remains challenging due to inherently poor soft-tissue contrast in non-enhanced scans [6]. Diffusible iodine-based contrast-enhanced CT (diceCT) has emerged as a transformative solution, enabling high-resolution three-dimensional visualization of internal structures without requiring physical dissection. Although micro-computed tomography (microCT) provides superior resolution for diceCT scanning, its application in cetacean research is often constrained by long scanning times, high operational costs, and, most importantly, limited availability in the veterinary and biological research settings typically accessible to stranding networks.

Clinical-grade CT scanners, which are widely available in medical and veterinary facilities, represent a practical yet underutilized alternative for post-mortem imaging when integrated with iodine-based contrast enhancement. While Lanzetti et al. [7] pioneered the application of diceCT in minke whale specimens, their results highlight opportunities to further refine soft-tissue enhancement protocols for marine mammal imaging, particularly for cetacean specimens. This need is particularly acute for the Narrow-Ridged Finless Porpoise (*Neophocaena asiaeorientalis*) (NRFP) - an IUCN Red List endangered species - given the exceptional rarity of well-preserved fetal specimens. Refining diceCT protocols for accessible imaging modalities is, therefore, critical to facilitate advancements in understanding NRFP developmental morphology, evolutionary adaptations, and pathological mechanisms.

This study presents the first application of clinical CT-based diceCT in an NRFP fetal specimen, providing a detailed protocol for our specimen preparation, scanning parameters, and 3D illustration. The aim of this study is to demonstrate the feasibility of clinical-grade CT as a potential alternative to microCT for high-fidelity soft-tissue imaging in precious cetacean fetal specimens.

## Method

In January 2023, an adult female NRFP was found stranded on the northern coast of Jeju Island, Republic of Korea (33°48’60.9"N 126°39’24.37"E) by local authorities and subsequently reported to the Marine Animal Imaging Laboratory^1^, an international marine animals stranding response and research network, for carcass retrieval and subsequent investigation. Necropsy revealed the specimen was pregnant. The fetal carcass, measuring 41 cm in length, was collected during the procedure and stored at −20 °C pending diffusible iodine-based contrast enhancement processing.

In November 2024, the fetal specimen was thawed at room temperature for 24 h to begin the diffusible iodine-based contrast enhancement process. The specimen was then immersed in 95% ethanol for two weeks for initial fixation and dehydration with one solution change on the second day. The specimen was stored in a double-sealed plastic container to prevent ethanol evaporation. The fetus was positioned with the tail region curled in a rounded posture to replicate its natural intrauterine position. According to the previous protocol [8], the staining solution was prepared by dissolving 14.78 g of reagent-grade iodine crystals (ACS Reagent, ≥ 99.8%, Sigma-Aldrich) in a mixture of 1.621 L of 95% ethanol and 0.579 L of distilled water, resulting in a 1% (w/v) iodine solution in 70% ethanol. Complete dissolution of iodine crystals was achieved through continuous stirring until no particulate matter remained visually observable. Throughout the staining period, the solution was replaced weekly to maintain a consistent iodine concentration and staining efficacy.

To evaluate the progression of iodine diffusion into the NRFP fetal specimen, CT scans were performed biweekly using a 16-row multislice spiral CT scanner (Alexion™, Canon Medical Systems) at the College of Veterinary Medicine, Kangwon National University, South Korea. All scans were performed with standardized parameters of 100 kVp, 300 mAs, and a section thickness of 0.3 mm. A field of view (FOV) of 180 mm was selected to ensure complete coverage of the specimen’s cross-sectional diameter. Reconstructed CT datasets were analyzed in Horos v3.3.6 (The Horos Project), a Digital Imaging and Communications in Medicine (DICOM)-compatible viewer. Complete staining was confirmed by the following criteria: (1) clear radiographic differentiation between adjacent tissue [9]; (2) variance of Hounsfield unit (HU) values of the specimen (optimal contrast); and (3) a plateau in the signal-to-noise ratio (SNR) among all scans within a homogeneous region of interest (ROI), specifically the liver parenchyma in the present study (optimal image quality) [10]. The SNR was calculated by defining the mean Hounsfield unit (MHU) of the segmented liver as the signal, and the standard deviation of the Hounsfield unit within that same ROI as the noise (σ) (SNR = MHU/σ).

For three-dimensional (3D) visualization, images were generated using the commercial segmentation platform - Eclipse v18.1 (Varian Medical Systems, Palo Alto, CA) through manual organ contouring of DICOM datasets, followed by post-processing with the open-source modeling software 3D Slicer (www.slicer.org).

Following successful diceCT imaging, the NRFP fetal specimen underwent destaining according to established protocols [8]. The specimen was immersed in 3% (w/v) sodium thiosulfate dissolved in 70% aqueous ethanol, formulated by first dissolving 66 g of reagent-grade sodium thiosulfate (Na₂S₂O₃, Sigma-Aldrich) in 0.579 L of distilled water, followed by gradual addition of 1.621 L of 95% ethanol with continuous stirring. The destaining solution was replaced biweekly to maintain chemical efficacy. Post-treatment destaining progress was monitored via clinical-grade CT (using parameters identical to the diceCT scanning protocol) monthly after the final diceCT scan until complete iodine clearance was confirmed by the return of mean Hounsfield unit (HU) values to baseline levels.

## Results

**Fig. 1:**
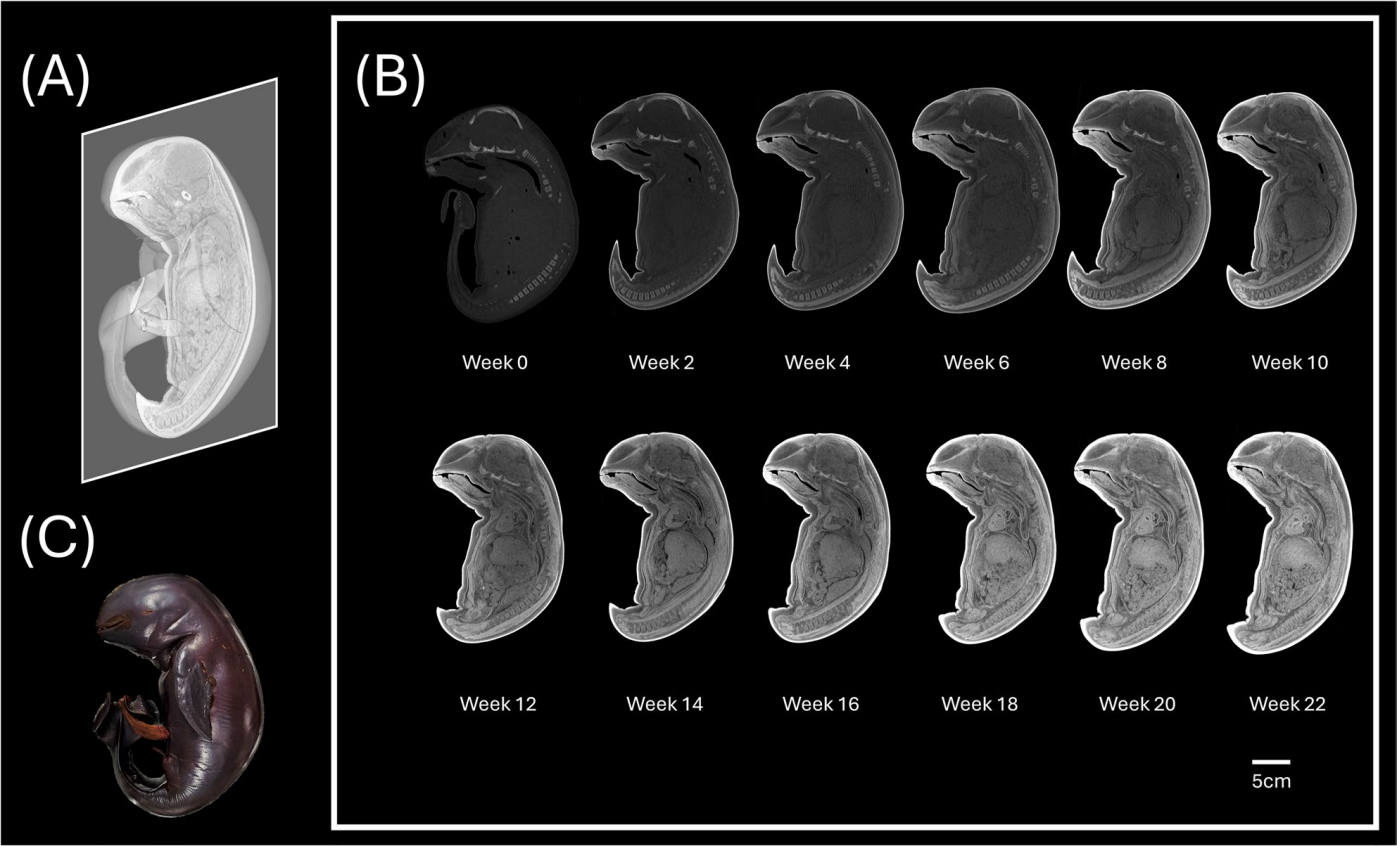
Diffusible iodine contrast-enhanced (diceCT) imaging of the NRFP fetal specimen. **A**-**B** Mid-sagittal views showing progressive enhancement of visceral organs with increasing staining duration (display window: WL 1000/WW 2600). **C** Post-staining specimen condition over 22 weeks, demonstrating tissue preservation integrity

**Fig. 2:**
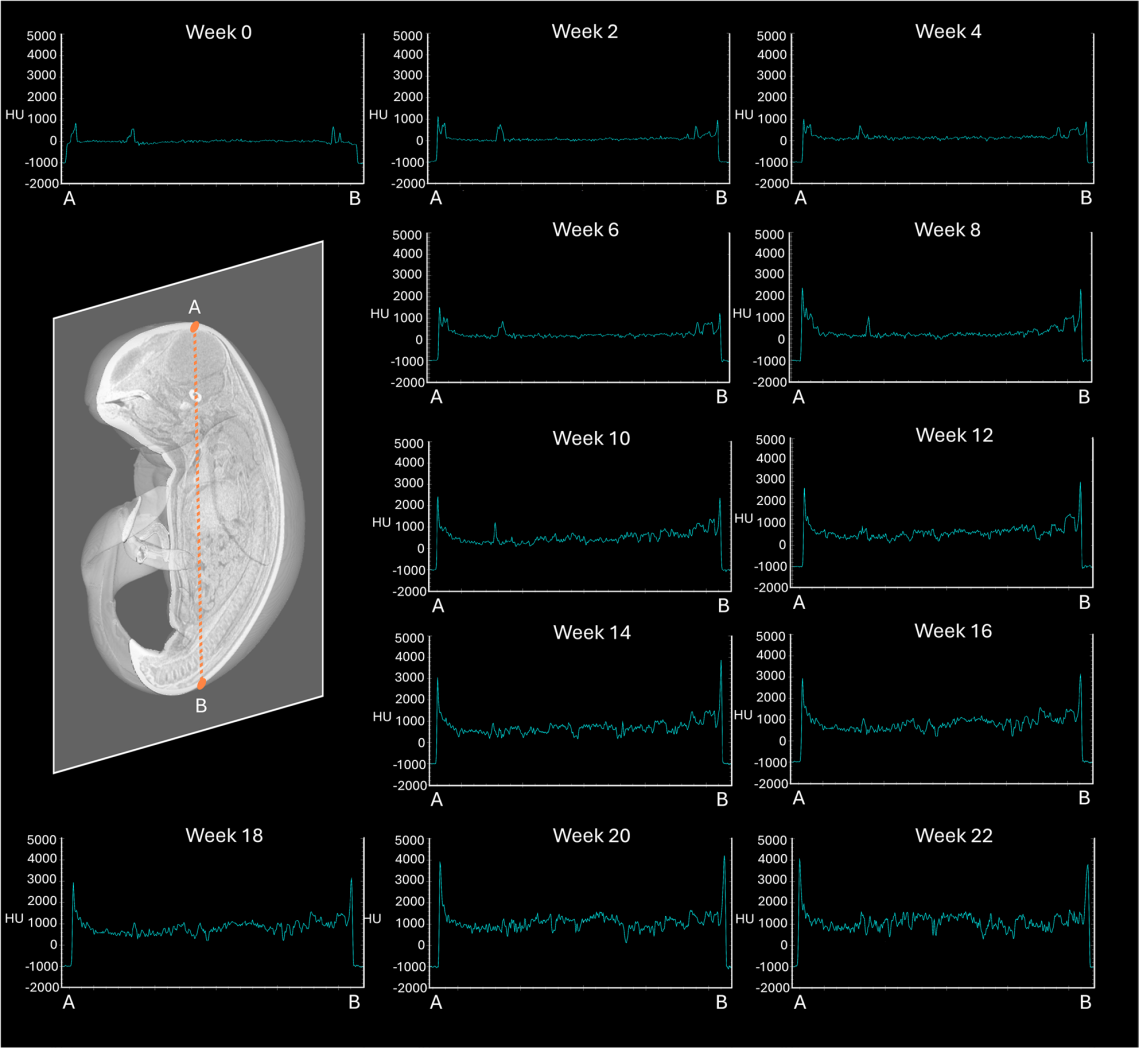
Plots showing the Hounsfield unit (voxel intensity value) corresponding to the line profile (orange lines) at each staining time point. The intensity range was set at −2000–5000 HU

**Fig. 3:**
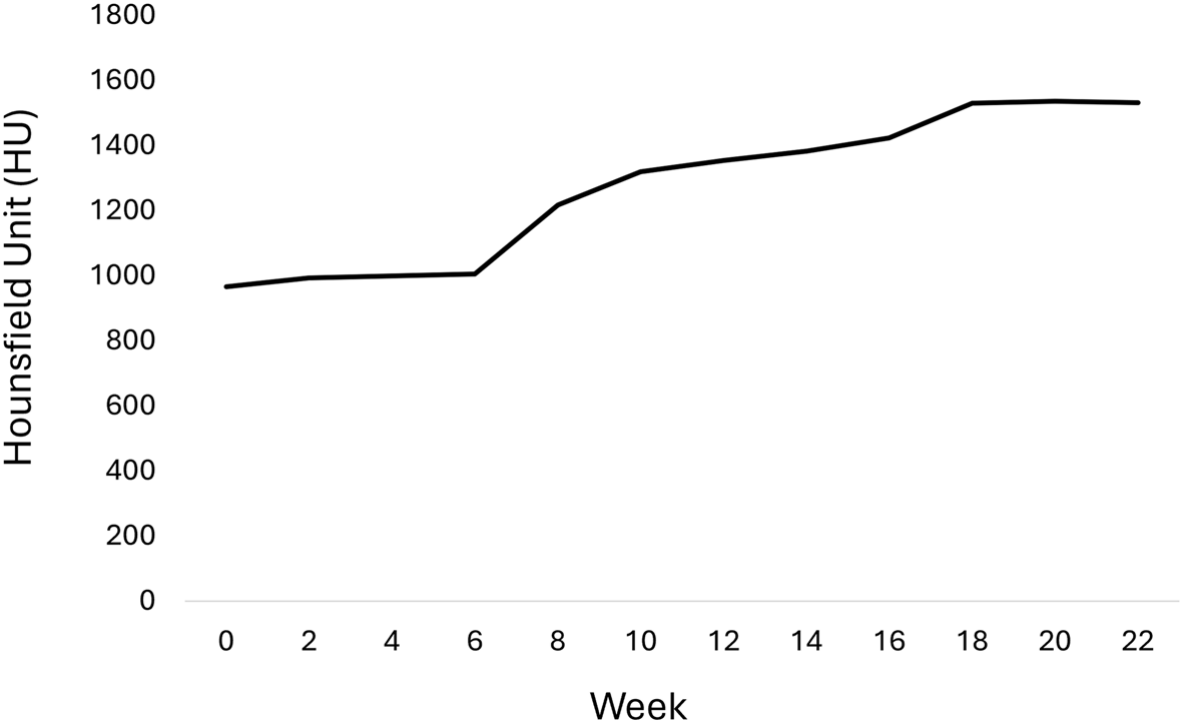
Progression of Hounsfield unit (HU) range in the NRFP fetal specimen over 22 weeks of iodine staining

**Fig. 4:**
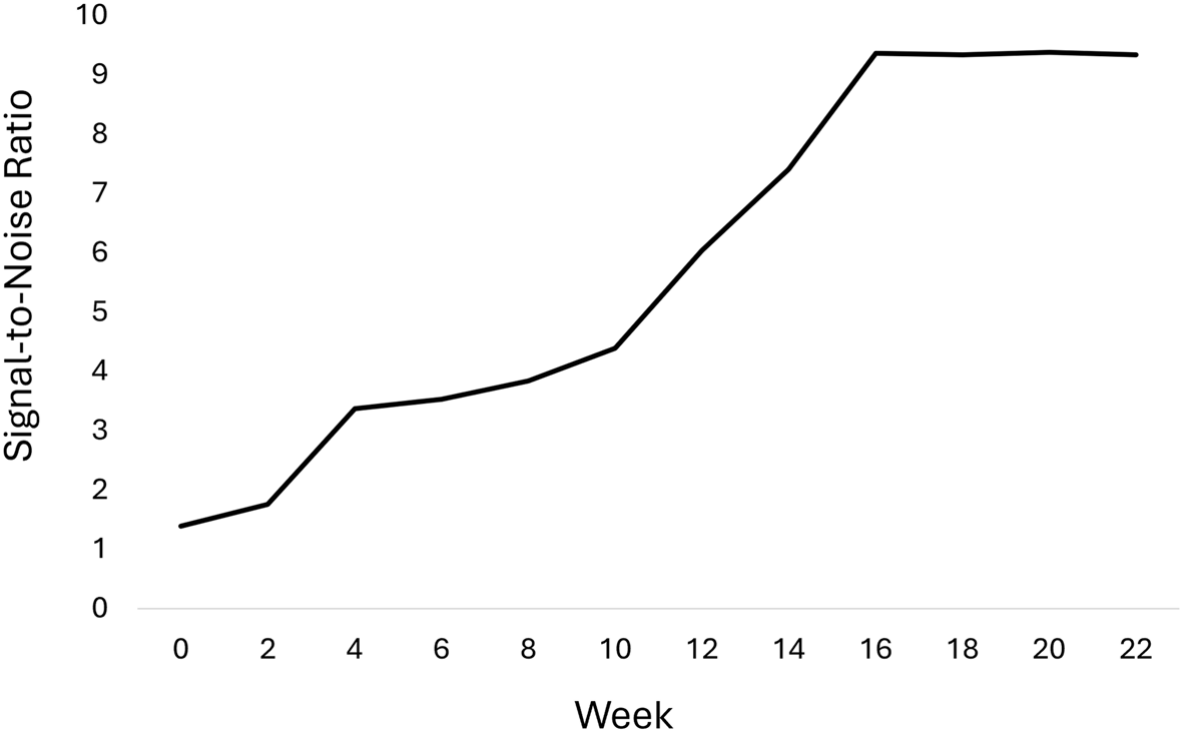
Temporal progression of liver signal-to-noise ratio over 22 weeks of iodine staining

**Fig. 5:**
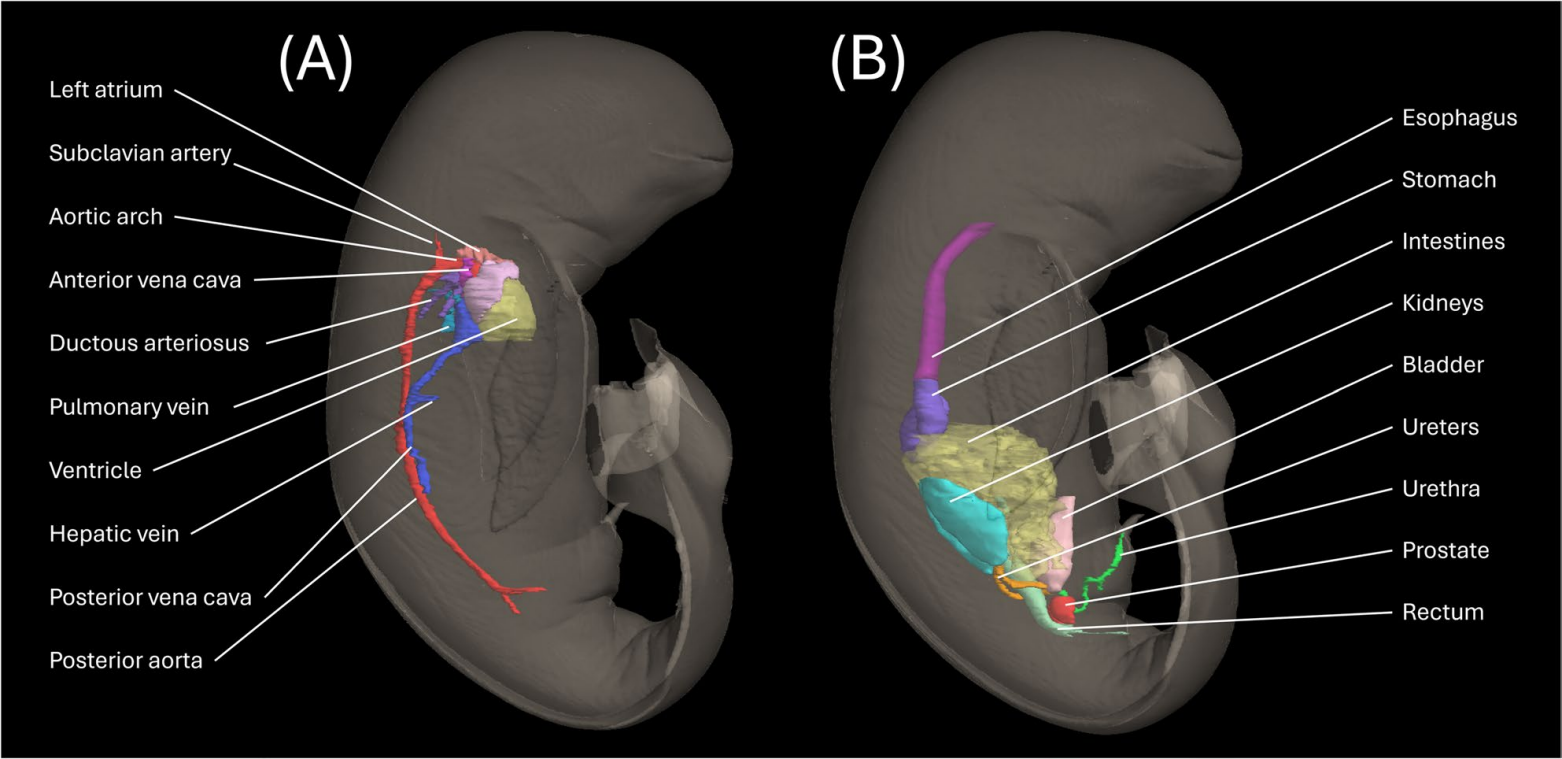
Three-dimensional reconstruction of the fetal (**A**) cardiovascular network, (**B**) gastrointestinal tract, and urogenital structures generated through manual contouring (right lateral view)

**Fig. 6:**
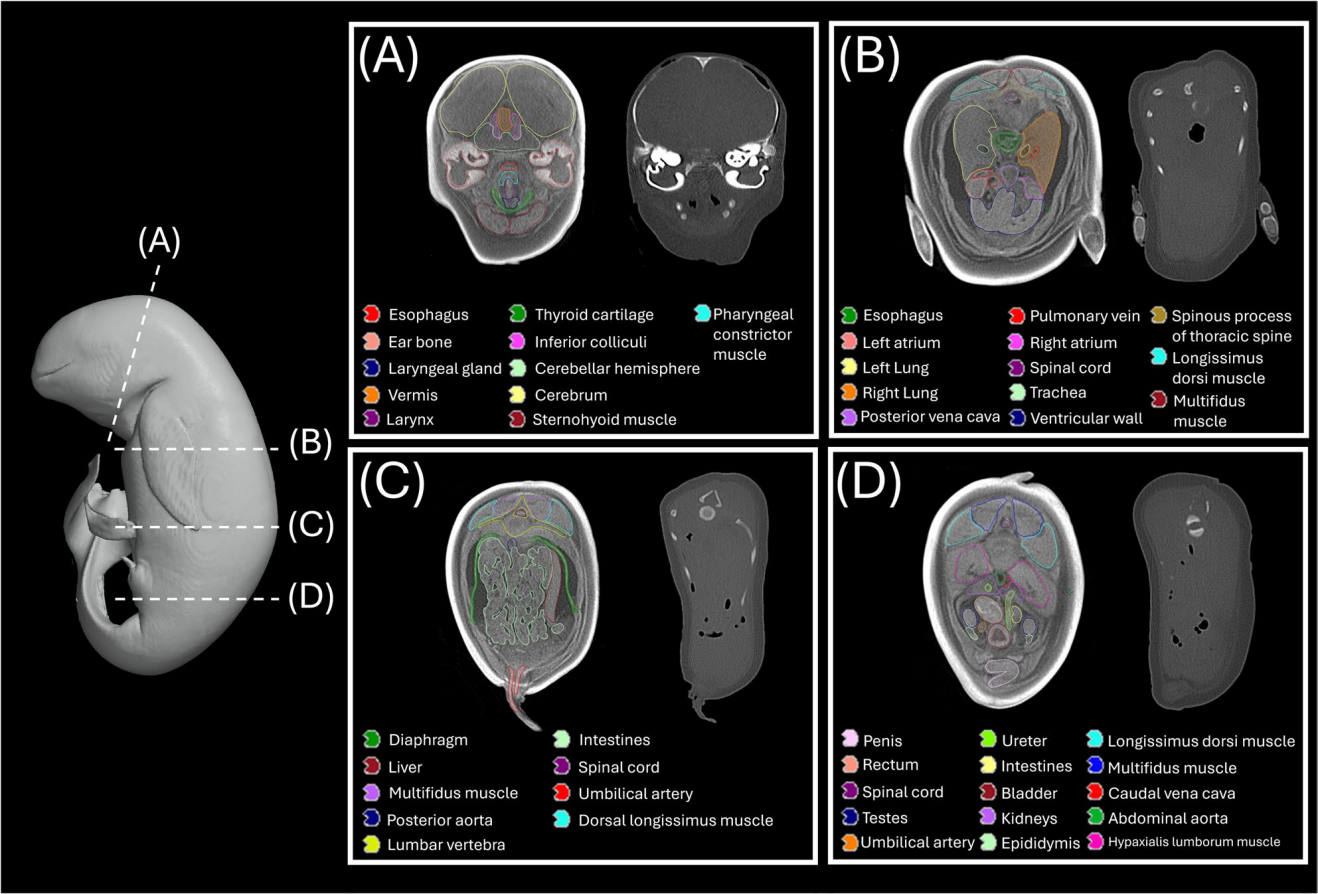
Transverse cross-sections of the diffusible iodine contrast-enhanced (diceCT) NRFP fetal specimen, demonstrating preserved organ topology in: (**A**) cranium, (**B**) thorax, (**C**) abdomen, and (**D**) pelvis. All structures are colored according to the anatomical key. Left panel: post-staining scan; right panel: pre-contrast baseline scan

In our study, serial biweekly CT imaging revealed progressive iodine enhancement in the NRFP fetal specimen over 22 weeks (11 scans), with contrast resolution evolving through three distinct phases: (1) initial surface penetration (Weeks 0–6), where staining was confined to superficial soft tissues; (2) intermediate visceral infiltration (Weeks 8–14), marked by improved but still incomplete organ delineation; and (3) final saturation (Weeks 16–22), achieving uniform enhancement with clear differentiation of major organs and vasculature (Fig. 1). The voxel intensity distribution showed progressively greater variation in Hounsfield unit (HU) values across organs from week 0 to week 22, reflecting gradual iodine uptake and enhanced tissue contrast. This variation reached a plateau at approximately week 18 (Figs. 2 and 3; Table 1). Concurrently, the signal-to-noise ratio (SNR) in the liver region of interest (ROI) plateaued at week 16, indicating optimal image quality was achieved at this earlier time point (Fig. 4; Table 2). High-resolution volumetric reconstructions (0.3 mm isotropic voxels) successfully captured the three-dimensional architecture of major organ systems, including the cardiovascular network, gastrointestinal tract, and urogenital structures (Fig. 5), with a substantial improvement in inter-tissue contrast resolution compared to native scans. The preservation of spatial relationships between organs throughout the staining process confirmed the non-destructive nature of this protocol (Fig. 6).

**Table 1:** Minimum, maximum, and range Hounsfield units of the NRFP fetal specimen over the 22-week staining period (excluding airspace and peripheral hyperenhanced regions)

Time point	Minimum HU	Maximum HU	HU range
Week 0	−163.02	803.90	966.92
Week 2	−185.53	808.52	994.05
Week 4	−180.77	818.32	999.09
Week 6	−182.03	824.36	1006.39
Week 8	6.39	1223.60	1217.21
Week 10	43.02	1362.39	1319.37
Week 12	154.43	1509.32	1354.89
Week 14	185.89	1569.36	1383.47
Week 16	185.96	1609.16	1423.2
Week 18	205.77	1736.35	1530.58
Week 20	205.30	1742.17	1536.87
Week 22	225.34	1756.78	1531.44

**Table 2:** SNR of the segmented liver in the NRFP fetal specimen over the 22-week staining period

Time point	Mean Hounsfield unit (MHU) (HU)	Standard deviation (σ)(HU)	Signal-to-noise ratio (SNR)
Week 0	31.55	22.69	1.39
Week 2	103.56	58.90	1.76
Week 4	161.06	47.74	3.37
Week 6	186.95	52.88	3.53
Week 8	333.69	86.96	3.84
Week 10	231.92	52.77	4.39
Week 12	546.15	90.37	6.04
Week 14	716.81	96.75	7.41
Week 16	936.61	100.08	9.36
Week 18	1116.11	119.47	9.34
Week 20	1244.16	132.64	9.38
Week 22	1391.53	148.99	9.34

## Discussion

This study establishes clinical-grade CT as a practical alternative to microCT for diceCT imaging of cetacean fetuses, presenting the first complete volumetric dataset of visceral organs in an NRFP fetus. While microCT systems can achieve higher resolution, their limited availability in marine research and stranding networks often precludes their use. Our clinical-grade CT protocol, utilizing widely available infrastructure, provides an efficient alternative while maintaining soft-tissue resolution sufficient for gross morphological analysis. This approach is particularly critical for handling rare specimens from strandings, where the unpredictable nature of events and the need to prevent tissue decomposition demand readily available and rapid imaging solutions.

Our findings demonstrate that clinical-grade diceCT provides high-fidelity, organ-scale morphological visualization sufficient for detailed gross anatomical analysis of cetacean fetal specimens. Based on our quantitative metrics, the period around week 18 represented an optimal state of enhancement, characterized by a maximized dynamic range in HU values and a peak in the signal-to-noise ratio, which provided excellent tissue contrast and image quality for morphological interpretation. This protocol facilitates the structure delineation of major anatomical systems, such as the cardiovascular branching patterns and intestinal stratification, with a clarity that enables comprehensive 3D modeling. The primary advantage of this approach lies in its practical utility. Given the widespread availability of clinical-grade CT scanners in veterinary and medical facilities [11], this method presents a highly accessible and logistically feasible alternative to more specialized imaging modalities, which are often associated with substantial costs and operational barriers for stranding networks. Therefore, clinical diceCT establishes itself as a pragmatic and powerful tool for institutions requiring detailed 3D morphological data of visceral organs, particularly in settings where access to advanced imaging is limited.

The NRFP fetal specimen in our study required substantially longer staining to reach optimal staining (18 weeks/126 days) than typical vertebrate models. Compared to fetal measurements reported for the Yangtze finless porpoise (*Neophocaena asiaeorientalis ssp. asiaeorientalis*) [12], the thoracic circumference (29.94 cm) and total body length (41 cm) of our fetal specimen may suggest a later gestational stage. While vertebrate models (e.g., avian, fish, and amphibian) achieve complete staining within days [13, 14], cetaceans present unique challenges due to their specialized integumentary system. The thick blubber layer and high lipid content in marine mammal skin may significantly retard iodine diffusion [15], as evidenced by pronounced contrast deposition in subcutaneous adipose tissue (Fig. 6). Similar constraints were observed by Lanzetti et al. [7], where a 70 cm humpback whale fetus achieved only subtle soft-tissue enhancement after 3 weeks of staining, even in mid-gestation [16]. This further demonstrates that marine mammal morphology necessitates extended protocols. These findings underscore the need for species- and size-adjusted approaches in diceCT, particularly for late-gestation specimens where tissue density peaks.

An important consideration in the present protocol is the potential for tissue shrinkage artifacts induced by ethanol-based fixation and staining. As noted by Dawood et al. [17], the use of iodine in ethanol solutions can cause soft-tissue shrinkage. This effect is consistent with the increased interstitial spaces observed around organs in our transverse cross-sections (Fig. 6B, C). Employing an ethanol-based protocol in the present study was a deliberate trade-off, prioritizing a critical objective over absolute morphometric fidelity: Specifically, for an exceptionally rare specimen of an endangered species, we selected a protocol that would preserve the potential for future biomolecular analyses (e.g., genetic, transcriptomic), which are often compromised by aldehyde-based fixatives like paraformaldehyde (PFA). While this choice may affect the accuracy of absolute morphometric data, it was deemed a necessary compromise to ensure effective contrast enhancement throughout the specimen and to maximize its future research value. Consequently, the primary aim of this study is to demonstrate the qualitative feasibility of clinical-grade diceCT for visualizing gross anatomical relationships. Future studies prioritizing exact morphometrics should consider the use of PFA fixation or explore recently developed shrinkage-mitigating staining solutions.

Notably, we identified regional heterogeneity in contrast uptake, with intracranial structures demonstrating lower observable differential iodine enhancement (Range (R): 482–1193 HU, standard deviation (SD) ± 101 HU) compared to other visceral organs: stomach (R: 348–1699 HU, SD: ± 211 HU), intestines (R: 198–1756 HU, SD: ± 261 HU), and kidneys (R: 389–1603 HU, SD: ± 211 HU) at week 18. This differential enhancement may directly correlate with tissue delineation capacity, as greater inter-tissue HU differences enable clearer structural definition in CT imaging. This pattern likely resulted from our whole-body staining approach - unlike previous studies that decapitated the specimens [18, 19] - suggesting the cranium may act as a diffusion barrier. This finding may indicate that iodine penetration occurs predominantly through soft tissue interfaces, with osseous structures significantly limiting solute exchange in intact specimens. For future studies requiring detailed visualization of specific organ systems, particularly the central nervous system, perfusion-based iodine delivery [20] could be explored to bypass diffusion barriers and achieve more targeted and homogeneous enhancement.

Alternatively, the observed differential enhancement could be influenced by thawing artifacts in the brain. Freeze-thaw cycles induce ice crystal formation, which may mechanically disrupt cellular membranes and microvasculature, altering vascular permeability and interstitial diffusion dynamics [21]. Such structural damage could disproportionately affect certain tissues—particularly those with high water content (e.g., brain parenchyma)—leading to heterogeneous contrast distribution. Additionally, partial autolysis during thawing may degrade tight junctions and extracellular matrices, further modifying tissue diffusion coefficients and solute retention. These artifacts could confound interpretations of iodine penetration. Therefore, to ensure optimal and uniform contrast enhancement, we recommend that future studies prioritize the use of fresh specimens where logistically feasible.

The NRFP fetal specimen utilized in this study has been completely destained through standardized protocols [8] and remains preserved at the Kangwon National University for future morphological investigations (Catalog No. NA23-0121_Fetus). This preserved specimen, along with the accompanying high-resolution DICOM datasets, provides enduring research value in multiple domains: (1) enabling comparative anatomical and developmental studies of NRFP through non-destructive means, (2) serving as educational resources for NRFP morphology, and (3) facilitating future methodological developments in contrast-enhanced imaging. The digital accessibility is particularly valuable for international collaborations, as demonstrated by our cross-institutional research teams (based in Hong Kong and South Korea), while maintaining the physical integrity of this rare specimen for long-term preservation. As the first complete diceCT dataset of an NRFP fetus obtained using clinical-grade CT, this resource establishes an important baseline for future studies of cetacean developmental anatomy.

This study is limited to a single specimen due to the exceptional rarity of obtainable NRFP fetal samples - a possible consequence of reduced population size [22] and low stranding frequencies of pregnant females. While sample size constraints are inherent to cetacean developmental research, we acknowledge that this limitation could affect the generalizability of findings. Future multi-institutional collaborations through international stranding networks may help mitigate this challenge by facilitating specimen aggregation.

## Conclusion

The present study has demonstrated that clinical-grade CT may provide a practical and accessible alternative to microCT for gross morphological studies of cetacean fetuses, leveraging widely available medical imaging infrastructure for diceCT applications. This approach significantly expands opportunities for morphological research on endangered species like the NRFP, particularly in resource-limited settings or stranding response scenarios where advanced imaging systems are unavailable. The preserved specimens and open-access DICOM datasets can further serve as important resources for future comparative and conservation-focused studies.

## Data Availability

CT images and specimen data are available upon request from AHLY (yhladams@gmail.com) and SWK (sangwhakim@kangwon.ac.kr), respectively.

## Footnotes

1 Marine Animal Imaging Laboratory - http://www.marine-animal-imaging-lab.org/.

## References

[1] Yuen AHL, Kim SW, Lee SB, Lee S, Lee YR, Kim SM, et al. Radiological investigation of gas embolism in the East Asian finless porpoise (*Neophocaena asiaeorientalis sunameri*). Front Mar Sci. 2022;9:711174.

[2] Lee SB, Yuen AHL, Lee YM, Kim SW, Kim S, Poon CTC, et al. Adhesive bowel obstruction (ABO) in a stranded narrow-ridged finless porpoise (*Neophocaena asiaeorientalis sunameri*). Animals. 2023;13(24):3767. 38136803 10.3390/ani13243767 PMC10741132

[3] Yuen AHL, Lee SB, Kim SW, Lee YM, Kim DG, Poon CTC, et al. Fatal upper aerodigestive tract obstruction in an East Asian finless porpoise (*Neophocaena asiaeorientalis sunameri*): findings in post-mortem computed tomography. Forensic Sci Med Pathol. 2024;20(2):644–51. 37831312 10.1007/s12024-023-00732-0

[4] Yuen AHL, Kim SW, Lee K, Lee YM, Lee SB, Kim MJ, et al. First report of kyphoscoliosis in the narrow-ridged finless porpoises (*Neophocaena asiaeorientalis*): findings from congenital and degenerative cases comparison using post-mortem computed tomography. Vet Med Sci. 2024;10(2):e31386. 38456337 10.1002/vms3.1386 PMC10921363

[5] Lee SB, Yuen AHL, Kim S, Jung WJ, Kim DG, Kim SW, et al. Ingestion of fishing gear and *Anisakis* sp. infection in a beached Indo-Pacific finless porpoise (*Neophocaena phocaenoides*) in the Jeju Island, Republic of korea: findings from post-mortem computed tomography and necropsy. BMC Vet Res. 2024;20(1):232. 38802879 10.1186/s12917-024-04090-z PMC11129503

[6] Faulwetter S, Vasileiadou A, Kouratoras M, Dailianis T, Arvanitidis C. Micro-computed tomography: introducing new dimensions to taxonomy. ZooKeys. 2013;263:1–45. 10.3897/zookeys.263.4261 PMC3591762 23653515

[7] Lanzetti A. Prenatal developmental sequence of the skull of minke whales and its implications for the evolution of mysticetes and the teeth-to-baleen transition. J Anat. 2019;235(4):725–48. 31216066 10.1111/joa.13029 PMC6742893

[8] Lanzetti A, Ekdale EG. Enhancing CT imaging: a safe protocol to stain and de-stain rare fetal museum specimens using diffusible iodine-based staining (diceCT). J Anat. 2021;239(1):228–41. 33665841 10.1111/joa.13410 PMC8197942

[9] Gignac PM, Kley NJ, Clarke JA, Colbert MW, Morhardt AC, Cerio D, et al. Diffusible iodine-based contrast-enhanced computed tomography (diceCT): an emerging tool for rapid, high-resolution, 3-D imaging of metazoan soft tissues. J Anat. 2016;228(6):889–909. 26970556 10.1111/joa.12449 PMC5341577

[10] Docter D, Timmerman M, Dawood Y, Hagoort J, Lobe N, van Heurn E, et al. Scaling up contrast-enhanced micro-CT imaging: optimizing contrast and acquisition for large ex-vivo human samples. Forensic Imaging. 2024;37:200596.

[11] Gielen I, Kromhout K, Gavin P, Van Ham L, Polis I, van Bree H. Agreement between low-field MRI and CT for the detection of suspected intracranial lesions in dogs and cats. J Am Vet Med Assoc. 2013;243(3):367–75. 23865879 10.2460/javma.243.3.367

[12] Zeng X, Chunyang L, Hao Y, Wang D, et al. Pregnancy diagnosis and fetal monitoring in Yangtze finless porpoises. Endang Species Res. 2022;47:291–6.

[13] Early CM, Morhardt AC, Cleland TP, Milensky CM, Kavich GM, James HF. Chemical effects of DiceCT staining protocols on fluid-preserved avian specimens. PLoS One. 2020;15(9):e0238783. 32946473 10.1371/journal.pone.0238783 PMC7500670

[14] Crowell HL, Nagesan RS, Davis Rabosky AR, Kolmann MA. Differential performance of aqueous- and ethylic-Lugol’s iodine stain to visualize anatomy in µCT-scanned vertebrates. J Anat. 2025;245(5):678–84. 10.1111/joa.14148 PMC11996703 39323056

[15] Gignac PM, Kley NJ. Iodine-enhanced micro-CT imaging: methodological refinements for the study of the soft-tissue anatomy of post-embryonic vertebrates. J Exp Zool B Mol Dev Evol. 2014;322(3):166–76. 24482316 10.1002/jez.b.22561

[16] van Aswegen M, Szabo A, Currie JJ, Stack SH, West KL, Hofmann N, et al. Energetic cost of gestation and prenatal growth in humpback whales. J Physiol. 2025;603:529–50. 39661448 10.1113/JP287304

[17] Dawood Y, Hagoort J, Siadari BA, Ruijter JM, Gunst QD, Lobe NHJ, et al. Reducing soft-tissue shrinkage artefacts caused by staining with lugol’s solution. Sci Rep. 2021;11(1):19781. 34611247 10.1038/s41598-021-99202-2 PMC8492742

[18] Li Z, Ketcham RA, Yan F, Maisano JA, Clarke JA. Comparison and evaluation of the effectiveness of two approaches of diffusible iodine-based contrast-enhanced computed tomography (diceCT) for avian cephalic material. J Exp Zool B Mol Dev Evol. 2016;326(6):352–62. 27511594 10.1002/jez.b.22692

[19] Green AL, Cowell EC, Carr LM, Hemsley K, Sherratt E, Collins-Praino LE, et al. Application of DiceCT to study the development of the Zika virus-infected mouse brain. Viruses. 2024;16(8):1330. 39205304 10.3390/v16081330 PMC11358961

[20] Gray JA, Gignac PM, Stanley EL. The first full body diffusible iodine-based contrast-enhanced computed tomography dataset and teaching materials for a member of the testudines. Anat Rec. 2024;307(3):535–48. 10.1002/ar.25282 37409685

[21] Hajal C, Offeddu GS, Shin Y, Zhang S, Morozova O, Hickman D, et al. Engineered human blood-brain barrier microfluidic model for vascular permeability analyses. Nat Protoc. 2022;17(1):95–128. 34997242 10.1038/s41596-021-00635-w

[22] Li Y, Cheng Z, Zuo T, Niu M, Chen R, Wang J. Distribution and abundance of the East Asian finless porpoise in the coastal waters of Shandong Peninsula, yellow Sea, China. Fishes. 2023;8(8):410.

